# Supplementary material for: Prevalence and Severity of Pelvic Floor Disorders during Pregnancy: Does the Trimester Make a Difference?
Source: Healthcare (Basel). 2023 Apr 11;11(8):1096. doi: 10.3390/healthcare11081096 (PMC10137441; doi:10.3390/healthcare11081096)
Supplement: Supplementary file 1 [file healthcare-11-01096-s001.zip › healthcare-2320409-supplementary.pdf]

Beckenboden-Fragebogen
für Schwangere und Frauen nach Geburt

MODUL RISIKOFAKTOREN **RISIKO**

Größe<br>□□□ cm	Gewicht<br>□□□ kg	Gewicht vor Schwangerschaft<br>Für BMI □□□ kg	BMI □□,□	☐ > 25
Alter			□□ Jahre	☐ > 35
Gibt es in Ihrer Familie blutsverwandte Frauen mit Harninkontinenz, Stuhlinkontinenz, Absinken der Beckenorgane?		☐ nein	☐ weiß nicht	☐ ja
Rauchen Sie?		☐ nein	☐ habe aufgehört	☐ ja
Können Sie Ihren Beckenboden gezielt anspannen?		☐ ja	☐ weiß nicht	☐ nein

Blasenfunktion

1. Wie häufig lassen Sie tagsüber Wasser? Pollakisurie	0 alle 3 Stunden	1 alle 2 Stunden	2 einmal pro Stunde	3 häufiger
2. Wie häufig erwachen Sie nachts, weil Sie Urin lassen müssen? Nykturie	0 0 – 1x	1 2x	2 3x	4 mehr als 3x
3. Verlieren Sie Urin im Schlaf? Enuresis nocturna	0 niemals	1 manchmal - weniger als einmal pro Woche	2 häufig – einmal oder mehr pro Woche	3 meistens - täglich
4. Ist der Harndrang so stark, dass Sie sofort zur Toilette eilen müssen? Imp Harndrang	0 niemals	1 manchmal - weniger als einmal pro Woche	2 häufig – einmal oder mehr pro Woche	3 meistens - täglich
5. Verlieren Sie bei plötzlichem sehr starken Harndrang Urin, bevor Sie die Toilette erreicht haben? Drangink	0 niemals	1 manchmal - weniger als einmal pro Woche	2 häufig – einmal oder mehr pro Woche	3 meistens - täglich
6. Verlieren Sie Urin beim Husten, Niesen, Lachen, Heben oder Sport? Belastungsink	0 niemals	1 manchmal - weniger als einmal pro Woche	2 häufig – einmal oder mehr pro Woche	3 meistens - täglich
7. Ist Ihr Harnstrahl schwach, verlangsamt oder verlängert? Harnstrahl	0 niemals	1 manchmal - weniger als einmal pro Woche	2 häufig – einmal oder mehr pro Woche	3 meistens - täglich
8. Haben Sie das Gefühl richtig einzuschätzen, wie voll Ihre Blase ist? Aff Vesica	0 ja - immer	1 meistens	2 manchmal	3 nein - niemals
9. Haben Sie das Gefühl, Ihre Blase nicht vollständig entleeren zu können? Restharn	0 niemals	1 manchmal - weniger als einmal pro Woche	2 häufig – einmal oder mehr pro Woche	3 meistens - täglich
10. Müssen Sie pressen, um Urin lassen zu können? Pressen	0 niemals	1 manchmal - weniger als einmal pro Woche	2 häufig – einmal oder mehr pro Woche	3 meistens - täglich
11. Tragen Sie Slipeinlagen oder Binden wegen eines Urinverlustes? Vorlagen	0 niemals	1 manchmal - nur als Prophylaxe	2 häufig – beim Sport / bei Erkältung	3 meistens - täglich

© Georg Thieme Verlag KG · DOI 10.1055/s-0043-102693 · Geburtsh Frauenheilk · Metz M et al.

12. Schränken Sie Ihre Trinkmenge ein, um Urinverlust zu vermeiden? Trinkverhalten	0 niemals	1 manchmal - weniger als einmal pro Woche	2 häufig – einmal oder mehr pro Woche	3 meistens - täglich	
13. Haben Sie ein Brennen, Ziehen oder Schmerzen beim Wasserlassen? Dysurie	0 niemals	1 manchmal - weniger als einmal pro Woche	2 häufig – einmal oder mehr pro Woche	3 meistens - täglich	
14. Wie häufig haben Sie Harnwegsinfekte? HWI	0 selten oder nie	1 1-3x pro Jahr	2 4-12x pro Jahr	3 1 oder mehr/Monat	
15. Beeinträchtigt der Urinverlust Ihr tägliches Leben? (z.B. Sport, Beruf, Einkauf, Ausgehen) QoL	☐ nicht zutreffend habe keine Symptome	0 überhaupt nicht	1 ein wenig	2 ziemlich	3 sehr
16. Wie sehr stören Sie Ihre Blasensymptome? Leidensdruck Blase	☐ nicht zutreffend habe keine Symptome	0 überhaupt nicht	1 ein wenig	2 ziemlich	3 sehr

Darmfunktion

1. Wie häufig haben Sie Stuhlgang? Frequenz	0 alle 3 Tage bis täglich	1 mehr als 1x täglich	1 alle 3 Tage oder seltener	2 weniger als einmal/Woche	
2. Wie ist die Konsistenz Ihres Stuhls normalerweise beschaffen? Konsistenz	0 weich oder geformt	1 verschieden	1 sehr hart	2 dünn/breiig	
3. Pressen Sie beim Stuhlgang sehr stark? Pressen	0 niemals	1 manchmal - weniger als einmal pro Woche	2 häufig – einmal oder mehr pro Woche	3 meistens - täglich	
4. Leiden Sie unter Verstopfungen? Obstipation	0 niemals	1 manchmal - weniger als einmal pro Woche	2 häufig – einmal oder mehr pro Woche	3 meistens - täglich	
5. Entweichen Ihnen Winde oder Blähungen, ohne dass Sie sie zurückhalten können? Flatusinkontinenz	0 niemals	1 manchmal - weniger als einmal pro Woche	2 häufig – einmal oder mehr pro Woche	3 meistens – täglich	
6. Bekommen Sie Stuhldrang, den Sie nicht zurückdrängen können? Imp Stuhlldrang	0 niemals	1 manchmal - weniger als einmal pro Woche	2 häufig – einmal oder mehr pro Woche	3 meistens – täglich	
7. Finden Sie auf Ihrer Wäsche oder auf Vorlagen Verfärbungen durch Stuhl? Stuhlschmieren	0 niemals	1 manchmal - weniger als einmal pro Woche	2 häufig – einmal oder mehr pro Woche	3 meistens – täglich	
8. Verlieren Sie oder entweicht Ihnen versehentlich Stuhl? Stuhlink	0 niemals	1 manchmal - weniger als einmal pro Woche	2 häufig – einmal oder mehr pro Woche	3 meistens – täglich	
9. Haben Sie das Gefühl, den Darm nicht vollständig entleeren zu können? Entleerungsstörungen	0 niemals	1 manchmal - weniger als einmal pro Woche	2 häufig – einmal oder mehr pro Woche	3 meistens - täglich	
10. Beeinträchtigen die Symptome Ihr tägliches Leben? (Tagesplanung, Sport, Beruf, Einkauf, Ausgehen) LQ	☐ nicht zutreffend – habe keine Symptome	0 überhaupt nicht	1 ein wenig	2 ziemlich	3 sehr
11. Wie sehr stören Sie Ihre Darmsymptome? Leidensdruck Darm	☐ nicht zutreffend – habe keine Symptome	0 überhaupt nicht	1 ein wenig	2 ziemlich	3 sehr

Senkung

1. Haben Sie ein Fremdkörpergefühl in der Scheide? Fremdk	0 niemals	1 manchmal - weniger als einmal pro Woche		2 häufig – einmal oder mehr pro Woche	3 meistens – täglich
2. Haben Sie das Gefühl, dass sich Ihre Scheide oder Gebärmutter abgesenkt haben? DeszGefühl	0 niemals	1 manchmal - weniger als einmal pro Woche		2 häufig – einmal oder mehr pro Woche	3 meistens – täglich
3. Haben Sie das Gefühl, dass sich beim Heben, Gehen oder Rennen Ihre Scheide oder Ihre Gebärmutter absenken? DeszBelastung	0 überhaupt nicht	1 ein wenig		2 ziemlich	3 sehr
4. Beeinträchtigen diese Symptome Ihr tägliches Leben? (z.B. Sport, Beruf, Einkauf, Ausgehen) LQ	☐ nicht zutreffend – habe keine Symptome	0 überhaupt nicht	1 ein wenig	2 ziemlich	3 sehr
5. Wie sehr stört Sie Ihre Senkung? LD Desz	☐ nicht zutreffend – habe keine Symptome	0 überhaupt nicht	1 ein wenig	2 ziemlich	3 sehr

Sexualität

Sind Sie sexuell aktiv? Sex Aktiv	☐ gar nicht	☐ selten	☐ regelmäßig		
Falls Sie keinen Verkehr haben, warum nicht? Abstinent weil	☐ kein Partner	☐ Partner hat Problem/impotent	☐ empfinde keine Erregung/keine Lust		☐ Sex ist mir unangenehm, weil ……………………….
Haben Sie sexuelle Erfahrungen gemacht, die Sie sehr belasten? SexTrauma	☐ nein				☐ ja
1. Wird Ihre Scheide während des Verkehrs ausreichend feucht? Lubrikation	0 ja				1 nein
2. Wie ist das Gefühl in der Scheide während des Verkehrs? AfferenzVag	0 fühle viel	1 fühle wenig	2 fühle nichts		3 habe Schmerzen
3. Denken Sie, dass Ihre Scheide zu schlaff oder weit ist? VagWeite	0 nein - niemals	1 manchmal	2 häufig		3 immer
4. Denken Sie, dass Ihre Scheide zu eng oder straff ist? Vaginismus	0 nein – niemals	1 manchmal	2 häufig		3 immer
5. Haben Sie Schmerzen während des Verkehrs? Dyspareunie	0 nein – niemals	1 manchmal	2 häufig		3 immer
6. Falls Sie Schmerzen während des Verkehrs haben, wo sind die Schmerzen?	1 am Scheideneingang		1 tief innerlich / im Becken		2 beides
7. Verlieren Sie unbeabsichtigt Urin oder Stuhl beim Sex? Koitale Inkontinenz	0 nein – niemals	1 manchmal	2 häufig		3 immer
8. Beeinträchtigen diese Symptome Ihre Sexualität? LQ	☐ nicht zutreffend – habe keine Symptome	0 überhaupt nicht	1 ein wenig	2 ziemlich	3 sehr
9. Wie sehr stören Sie diese Symptome? Leidensdruck Sex	☐ nicht zutreffend – habe keine Symptome	0 überhaupt nicht	1 ein wenig	2 ziemlich	3 sehr

Score (Hier bitte nichts eintragen)

**Blasenfunktion**	Fragen 1- 14	Score ☐☐ / 44 =	+		x 10 =
**Darmfunktion**	Fragen 1- 9	Score ☐☐ / 27 =	+		x 10 =
**Senkung**	Fragen 1- 3	Score ☐☐ / 11 =	+		x 10 =
**Sexualität**	Fragen 1- 7	Score ☐☐ / 19 =	+		x 10 =
**Blasenscore + Darmscore + Senkungsscore + Sexscore =**					

Postpartales Modul

Risiko

Wie viele Kinder haben Sie geboren?	☐☐	Bei wie vielen Geburten wurde eine Saugglocke zur Hilfe genommen?			☐
Bei wie vielen Geburten wurde ein Kaiserschnitt gemacht?	☐	Bei wie vielen Geburten wurde eine Zange zur Hilfe genommen?			☐
Wieviel wog Ihr schwerstes Kind bei der Geburt?		☐☐☐☐ g			☐ > 4000g
Hatten Sie bei einer Ihrer Geburten Schließmuskel- oder Darmverletzungen (Dammriss 3. oder 4. Grades)?		☐ nein			☐ ja
Hatten Sie nach der Geburt Schmerzen im Scheiden-, Damm- oder Darmbereich?		☐ nein			☐ ja
Haben Sie das Gefühl, die Geburtsschmerzen bzw. die Schmerzen nach der Geburt verarbeitet zu haben?		☐ ja	☐ größtenteils	☐ ein wenig	☐ nein
Haben Sie das Gefühl, die Ängste, die Sie unter der Geburt hatten, verarbeitet zu haben?		☐ ja	☐ größtenteils	☐ ein wenig	☐ nein

© Georg Thieme Verlag KG · DOI 10.1055/s-0043-102693 · Geburtsh Frauenheilk · Metz M et al.
